# From Mandate to Choice: How Voluntary Mask Wearing Shapes Interpersonal Distance Among University Students After COVID-19

**DOI:** 10.3390/healthcare13161956

**Published:** 2025-08-09

**Authors:** Yi-Lang Chen, Che-Wei Hsu, Andi Rahman

**Affiliations:** 1 Department of Industrial Engineering and Management, Ming Chi University of Technology, New Taipei 243303, Taiwan; m10218009@mail2.mcut.edu.tw (C.-W.H.); m09218051@mail2.mcut.edu.tw (A.R.); 2 Quanta Computer Inc., Taoyuan 33377, Taiwan; 3 Department of Industrial Engineering, Andalas University, Padang 25175, Indonesia

**Keywords:** interpersonal distance (IPD), mask-wearing choice, public health behavior, participant gender, target gender

## Abstract

**Background/Objectives:** As COVID-19 policies shift from government mandates to individual responsibility, understanding how voluntary protective behaviors shape social interactions remains a public health priority. This study examines the association between voluntary mask wearing and interpersonal distance (IPD) preferences in a post-mandate context, focusing on Taiwan, where mask wearing continues to be culturally prevalent. **Methods:** One hundred university students (50 males, 50 females) in Taiwan completed an online IPD simulation task. Participants adjusted the distance of a virtual avatar in response to targets that varied by gender and mask status. Mask-wearing status upon arrival was recorded naturally, without manipulation. A four-way ANOVA tested the effects of participant gender, participant mask wearing, target gender, and target mask wearing on the preferred IPD. **Results:** Voluntary mask wearing was more common among female participants (72%) than males (44%). Mask-wearing individuals maintained significantly greater IPDs, suggesting heightened risk perception, whereas masked targets elicited smaller IPDs, possibly due to social signaling of safety. Gender differences emerged in both protective behavior and spatial preferences, with females showing stronger associations between mask use and distancing behavior. **Conclusions:** These findings offer actionable insights into how voluntary behavioral adaptations continue to shape spatial interaction norms after mandates are lifted. The integration of real-time simulation and statistical modeling highlights the potential of digital behavioral tools to support culturally adaptive, person-centered public health strategies.

## 1. Introduction

The COVID-19 pandemic fundamentally reshaped human social behavior, with interpersonal distance (IPD) emerging as a central aspect of public health strategies [1,2]. IPD—defined as the physical space that individuals maintain between themselves and others during social interactions—has been widely used as an indicator of perceived risk and engagement in protective behaviors [3,4]. Examining how protective behaviors are associated with spatial preferences is essential for public health planning, as these associations may reflect underlying psychological processes that support adherence to health guidelines [5].

The relationship between mask wearing and IPD can be understood through several interconnected psychological frameworks. Risk compensation theory suggests that when individuals adopt one protective behavior, such as mask wearing, they may adjust other behaviors—like social distancing—accordingly [6]. Masks also function as visual cues that may signal health consciousness and perceived risk to others. Upon encountering a masked individual, people may interpret the behavior as indicating heightened caution, potentially prompting an increased distance, or as a sign of responsibility, possibly reducing the perceived threat and allowing closer proximity [7,8,9]. Protection motivation theory further proposes that protective behaviors are associated with both threat and coping appraisals, implying that voluntary mask wearers may perceive a greater threat and therefore prefer larger IPDs [10].

During the pandemic, numerous studies examined how mandated mask wearing was associated with changes in IPD, reporting mixed results across cultural and situational contexts [11,12,13,14,15,16]. However, these investigations primarily addressed behaviors influenced by governmental mandates rather than voluntary choices. As societies transition from mandate-driven to choice-driven protective practices, an important knowledge gap remains: how is voluntary mask wearing—reflecting personal risk assessment and health consciousness—related to IPD preferences in post-pandemic social interactions?

This question has important implications for public health policy. Gaining insights into how voluntary protective behaviors are associated with one another can help to inform future pandemic preparedness strategies. In addition, the continued presence of altered IPD preferences may be linked to long-term effects on social functioning and psychological well-being [17,18]. The transition from mandated to voluntary mask wearing offers a natural context to explore how individual differences in risk perception and cultural norms are related to spatial behavior.

Research on post-pandemic IPD has produced mixed findings, emphasizing the importance of distinguishing between mandated and voluntary protective behaviors. Welsch et al. [11] found that IPD preferences in Germany did not return to pre-pandemic levels even after restrictions were lifted, suggesting persistent behavioral adaptation. In contrast, Chen et al. [15] reported a rapid reduction in perceived IPD among young Taiwanese individuals following the removal of mask mandates, highlighting cultural and demographic variability in behavioral persistence. These contrasting results underscore the need to examine voluntary protective behaviors independently of compliance-driven responses, as voluntary behaviors may reflect more stable, intrinsic motivations that persist beyond external mandates. While existing studies offer useful insights into pandemic-era spatial behavior, few have explored how voluntary mask wearing—as a marker of intrinsic motivation rather than compliance—is associated with IPD in post-mandate contexts. The present study directly addresses this gap by examining the bidirectional relationship between voluntary protective choices and spatial preferences, offering insights into sustained behavioral adaptations that extend beyond policy enforcement.

Previous research has identified key demographic factors associated with protective behaviors and spatial preferences. Gender differences in health-related behaviors and COVID-19 risk perception have been consistently observed, with females generally exhibiting greater compliance and higher risk awareness [5,15,19,20]. Age-related variation in interpersonal space preferences has also been noted, with younger individuals displaying distinct spatial behavior patterns [21,22]. However, how voluntary mask wearing interacts with these demographic factors in post-mandate contexts remains insufficiently explored.

The distinction between mandated and voluntary protective behaviors is theoretically important, as voluntary behaviors are more likely to reflect intrinsic motivation, personal risk perception, and individual health beliefs rather than external compliance [23]. When individuals choose to wear masks voluntarily, this decision may signal a sustained perception of threat and a protective orientation that could also be associated with spatial behaviors, such as IPD preferences. However, existing research has not sufficiently examined this bidirectional relationship between voluntary mask adoption and spatial preferences—a relationship that may reflect the complex interplay between individual protective strategies and perceived social comfort.

This research addresses a critical need to understand how cultural norms and individual autonomy interact in shaping post-pandemic social adaptation. In post-mandate contexts, voluntary protective behaviors can serve as indicators of sustained risk awareness and social responsibility, making their examination essential in developing long-term public health strategies that uphold personal choice while supporting community well-being. This theoretical gap is particularly relevant in post-pandemic Taiwan, where voluntary mask wearing has become embedded in everyday social norms. Several factors appear to contribute to the continued use of masks in Taiwan and other East Asian societies. A long-standing tradition of mask wearing during respiratory illness seasons has been reinforced by experiences during the COVID-19 pandemic [24]. The cultural emphasis on collective well-being and social harmony has helped to normalize mask use as a symbol of mutual respect and health consciousness [25]. Additionally, positive experiences with mask wearing—particularly in crowded public spaces—have facilitated its incorporation into daily routines [26,27]. Taiwan’s post-mandate environment thus offers a unique context in which to explore how voluntary mask wearing has shifted from policy-driven behavior to a culturally integrated practice.

This study addresses key knowledge gaps by examining how voluntary mask-wearing behavior is associated with IPD preferences in a post-mandate context, with the goal of providing evidence-based insights for public health policy and spatial management. We aimed to explore how individuals’ voluntary mask-wearing choices relate to IPD preferences, assess the social signaling effects of encountering masked versus unmasked individuals, and identify gender-specific patterns that may inform actionable recommendations for public health practitioners and policymakers.

We recruited 100 university students (50 males, 50 females) and recorded their spontaneous mask-wearing behavior upon arrival at the experimental site, allowing for the naturalistic observation of personal protective decisions. By analyzing IPD preferences in response to virtual targets differing in gender and mask status, we examined how voluntary protective behaviors are related to spatial judgments in a post-mandate setting. This design enabled us to investigate both how individuals’ own mask-wearing statuses corresponded with their preferred IPDs and how exposure to masked versus unmasked targets influenced their spatial behavior.

Grounded in risk compensation theory, social signaling theory, and protection motivation theory, we proposed four testable hypotheses. We hypothesized that individuals who voluntarily wore masks would maintain larger IPDs than non-mask wearers, reflecting heightened risk perception and a broader protective orientation. Encountering masked targets was expected to result in reduced IPDs, as mask wearing may signal safety and social responsibility. We further hypothesized that gender would be associated with both voluntary mask-wearing prevalence and the strength of the relationship between protective behaviors and spatial preferences, with females expected to show higher mask adoption rates and stronger associations. Finally, we anticipated that these behavioral patterns would produce measurable effect sizes sufficient to inform evidence-based recommendations for public space design and health communication strategies.

## 2. Materials and Methods

This study employed an observational experimental design to examine how voluntary mask-wearing behavior is associated with IPD preferences in post-pandemic contexts. To capture natural protective behavior, participants’ mask-wearing statuses were recorded upon arrival without manipulation. They then completed an online IPD simulation task, adjusting the distance of a virtual avatar in response to targets that varied by gender and mask status. This approach allowed for the analysis of both how individuals’ own mask-wearing behavior corresponded with their spatial preferences and how masked versus unmasked targets influenced IPD judgments. In doing so, the study directly addressed the relationship between voluntary protective behaviors and emerging social spatial norms. Ethical approval was obtained from the Ethics Committee of Chang Gung University, Taiwan, and all procedures were conducted in accordance with the 2013 World Medical Association Declaration of Helsinki and relevant institutional guidelines.

### 2.1. Participants

A total of 100 participants—equally divided between males and females—were enrolled in an online test. All were undergraduate or graduate students who reported no cognitive or psychological impairments. The average (standard deviation) ages were 21.4 (2.2) years for males and 20.9 (1.8) years for females. All participants were right-handed and unfamiliar with the target individuals used in the simulation. Data collection was conducted in September 2023, following Taiwan’s phased lifting of mask mandates. This transition began on 17 April 2023, when the government removed most public mask-wearing requirements. Although certain settings, particularly healthcare facilities, retained mandates until 19 May 2024, mask wearing in most public spaces had largely shifted to a matter of personal choice during our data collection period. This timing enabled the study to examine voluntary mask-wearing behavior in a transitional social context—when external mandates had been lifted for most environments, but institutional requirements remained in select locations. Informed consent was obtained from all participants, including consent for the publication of identifying information and images in an open-access format.

While this sample offers valuable insights into young adult behavior within the Taiwanese context, the exclusive recruitment of university students from a single country may limit the generalizability of the findings to other populations, age groups, or cultural settings. The relatively homogeneous demographic profile—young, educated, and Taiwanese—should be taken into account when interpreting the results, as voluntary protective behaviors and spatial preferences may differ across socioeconomic, educational, and cultural backgrounds.

### 2.2. Experimental Setting

Although the pandemic had subsided, we employed an online test to collect IPD data, following the approach used by Chen and Rahman [5], to allow for comparison with prior studies. The online IPD measurement protocol gained widespread use during the pandemic and was adapted from the original paper-and-pencil methods developed by Hayduk [28] and Xiong et al. [29]. Our version of the test, widely accepted in both clinical and applied research settings [21], was implemented using Axure RP 11, a rapid prototyping tool (Version 11, Axure Software Solutions, San Diego, CA, USA).

During the test, participants used a computer cursor to move a virtual subject (avatar) toward a designated target. To avoid influencing distance judgments, the directional arrow between the avatars was hidden once movement began. No numerical cues were given regarding the distance between avatars; instead, participants relied solely on spatial perception as the avatar advanced. They were instructed to stop at a point that felt comfortable but had just begun to feel slightly uncomfortable—consistent with definitions used in prior studies [2,5,20,30,31,32]. The final avatar distance was converted to the psychological IPD using a 1:7.2 scale ratio. The starting point of 55.5 cm corresponded to an approximate real-world separation of 4 m between the participant and target [2,32]. Reliability was confirmed in a pilot study, which yielded a satisfactory intraclass correlation coefficient of 0.85 across repeated trials.

### 2.3. Targets

**Figure 1:**
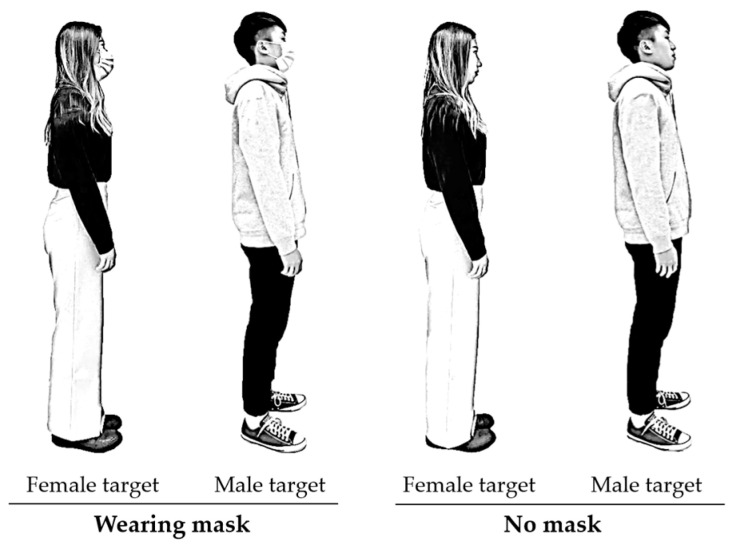
Images of the targets under different testing conditions (2 genders × 2 mask-wearing statuses), post-processed and manually redrawn to anonymize identities.

A male and a female, both 22 years old, were selected as target subjects, with respective heights of 176 cm and 160 cm. Both individuals were Taiwanese, ensuring demographic consistency with the participant population and cultural context of the study. Each subject was photographed wearing casual clothing with no accessories. Using a digital camera (Sony HDR-XR260; Sony, Tokyo, Japan), sagittal-view images were captured under four conditions—two based on gender and two on mask-wearing status—following the methodology used by Chen and Rahman [5], as illustrated in Figure 1. Throughout the image capture process, the subjects maintained neutral facial expressions. These photographs were then used as digital stimuli in the online test. To standardize the visual presentation, the images of the male and female targets were scaled to screen heights of 24.4 cm and 22.2 cm, respectively. The surgical masks worn in the masked conditions were plain blue and unembellished, representative of the typical face coverings used during the COVID-19 pandemic.

Several limitations related to the visual stimuli should be acknowledged. The use of only two target individuals with neutral expressions and standardized attire may not fully represent the diversity of social cues that influence IPD judgments in real-world settings. Moreover, the static nature of photographic stimuli—although beneficial for experimental control—differs from dynamic, face-to-face interactions, where movement, facial expressions, body language, and the situational context can influence spatial decision making. These limitations may have shaped participants’ perceptions of the social signals conveyed by mask wearing and could impact the ecological validity of the findings.

### 2.4. Procedure

The primary aim of this study was to examine how participants’ voluntary mask-wearing behavior was associated with their IPD preferences. Upon arrival at the experimental site, the experimenter visually recorded whether each participant was wearing a face mask. No instruction was given to wear or remove a mask, allowing for natural variations in protective behavior. Participants remained under observation throughout the session to ensure that those who arrived wearing a mask retained it during the entire experiment. No participant changed their mask status during testing.

**Figure 2:**
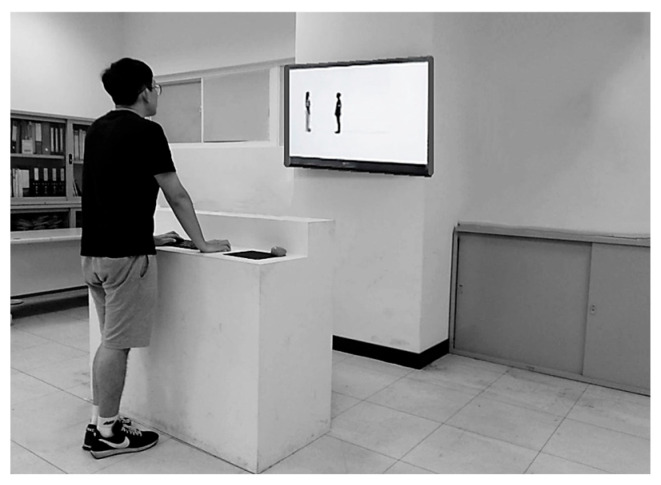
Schematic illustration of the experimental layout and testing procedure.

Before the session began, all participants underwent a health screening to confirm that they were asymptomatic for respiratory illnesses, including cold, fever, COVID-19, and related conditions. This screening ensured that mask-wearing decisions reflected voluntary protective behavior and perceived risk, rather than immediate health-related needs. After screening, standardized instructions were provided. Participants were told that they would use a computer mouse to move a side-profile avatar toward a target person shown on the screen and should stop at the point where they would begin to feel uncomfortable if the interaction were occurring in real life (Figure 2). They were instructed to imagine that the approaching avatar represented themselves, and the static image represented another person. This procedure was adapted from previous studies on virtual IPD assessment [2,28].

Each participant completed 12 trials, including three repeated measures for each of the four target conditions (2 genders × 2 mask statuses). Target presentations were randomized, and rest intervals of at least three minutes were included between trials to minimize fatigue or habituation effects. At the start of each trial, participants viewed frontal images of the target under all four conditions to facilitate the visualization of the scenario. They then performed the IPD task by adjusting the avatar’s horizontal position to the point just before discomfort. Minor final adjustments were allowed, and the system automatically recorded the chin-to-chin distance between avatars. These values were subsequently converted to real-world measurements using a 1:7.2 scaling ratio to calculate the psychological IPD.

### 2.5. Statistical Analysis

The independent variables in the test included participant gender, participant mask status, target gender, and target mask status, while the dependent variable was the interpersonal distance (IPD), measured in centimeters. Data were analyzed using SPSS 23.0 (IBM, Armonk, NY, USA), with the significance level (α) set at 0.05. Because participant mask status was nested within gender, an unbalanced four-way nested ANOVA was conducted to evaluate the effects of the independent variables on IPD. In this model, participant gender and participant mask status were treated as between-subject factors, while target gender and target mask status served as within-subject factors. Additionally, two separate three-way ANOVAs were performed for male and female participants, respectively, followed by post hoc comparisons using independent t-tests. Effect sizes were reported using η^2^ values. Prior to analysis, the Kolmogorov–Smirnov test confirmed that all numerical variables were normally distributed (all *p* > 0.05), and Levene’s test indicated homogeneity of variances across groups (all *p* > 0.05), supporting the use of ANOVA procedures. No missing data were identified, as all participants completed the full experimental protocol, with valid IPD measurements recorded for all 12 trials (three repetitions × four target conditions).

## 3. Results

**Figure 3:**
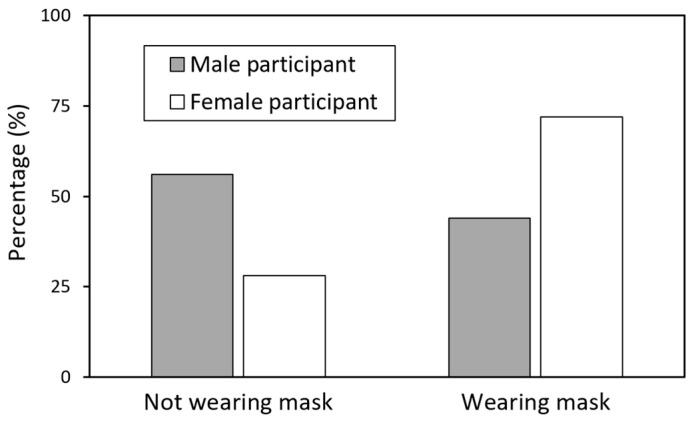
Proportions of mask wearing by gender. Female participants demonstrated significantly higher voluntary mask wearing (72%, *n* = 36) compared to males (44%, *n* = 22), χ^2^ = 7.84, *p* < 0.01. The overall prevalence of mask use among all participants was 58% (58 out of 100).

Figure 3 presents the proportions of male and female participants who wore masks upon arrival at the experimental site and throughout the experiment. A higher percentage of female participants (72%, *n* = 36) wore masks compared to male participants (44%, *n* = 22). Overall, 58% of the sample chose to wear masks despite the absence of a mandate, with the mask-wearing prevalence significantly higher among females than males.

**Figure 4:**
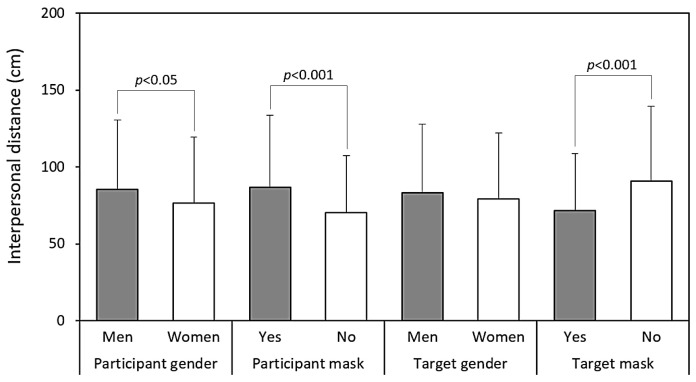
Main effects of the examined independent variables on interpersonal distance.

Table 1 summarizes the results of the four-way ANOVA conducted on IPD measurements. Participant gender (*p* < 0.05), participant mask status (*p* < 0.001), and target mask status (*p* < 0.001) were all significantly associated with differences in IPD, while target gender did not yield a significant main effect. Figure 4 illustrates these main effects, showing that male participants, masked individuals, and unmasked targets were associated with greater IPD values compared to their respective counterparts. In addition, Table 1 reveals a significant interaction between participant gender and target gender (*p* < 0.05), warranting further analysis to explore this relationship.

**Figure 5:**
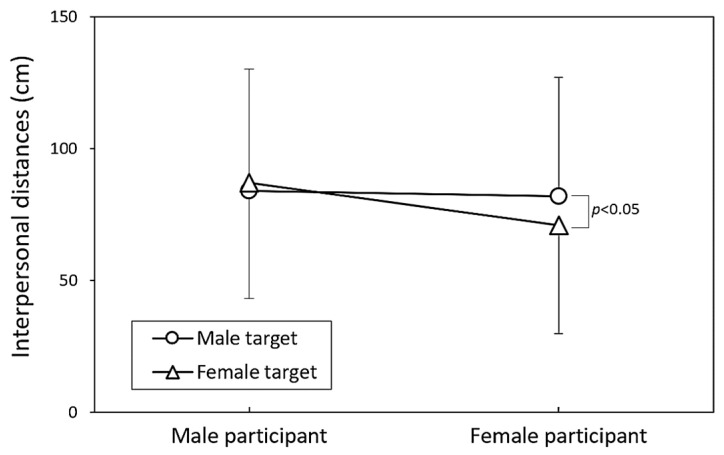
Comparison of interpersonal distance across participant genders in relation to target genders.

**Figure 6:**
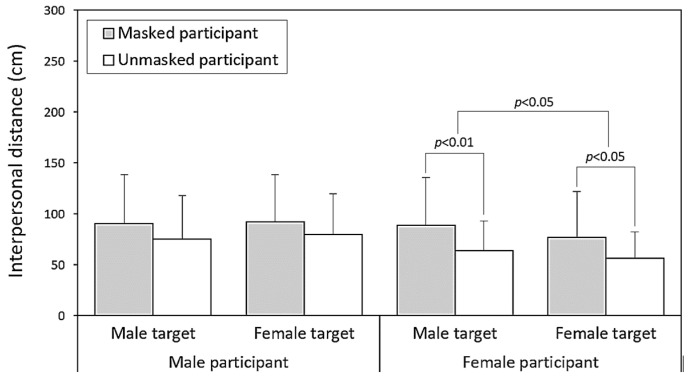
Pairwise comparisons of interpersonal distance values across participant mask-wearing statuses, based on independent t-tests conducted for each test condition.

Table 2 presents the results of the three-way ANOVA conducted separately for each participant gender. The effect of the target gender on IPD differed by participant gender, reaching significance for female participants (*p* < 0.05) but not for males (*p* = 0.430). Among female participants, the smallest IPD was observed when interacting with female targets, while the remaining three target combinations yielded nearly identical IPD values (Figure 5). Figure 6 further illustrates the significant differences in IPDs between masked and unmasked participants across both genders, with all paired comparisons reaching significance among female participants.

## 4. Discussion

This study provides new insights into how voluntary mask wearing is associated with IPD in post-pandemic contexts. Although mask wearing is now a matter of personal choice rather than public mandate, our findings suggest that it remains meaningfully related to social spacing behaviors—potentially reflecting both risk perception and comfort in social interactions. The observed patterns align with our study’s hypotheses, supporting possible associations between voluntary protective behaviors and IPD preferences, without implying direct causal relationships.

One key finding concerns the contrasting patterns associated with participant and target mask wearing. As shown in Table 1 and Figure 4, participants who wore masks tended to maintain larger IPDs than those who did not. This may reflect heightened risk perception or a general preference for increased personal space among individuals who choose to wear masks. Previous studies have noted that mask wearers often perceive themselves as more vulnerable to illness and consequently maintain greater distances from others [3,33]. In contrast, encountering a masked target was associated with a reduced IPD (71.6 cm vs. 90.6 cm), suggesting that masks may also function as prosocial signals that convey safety or trustworthiness [3,8]. This dual role—as both a protective barrier and a social signal—is consistent with earlier research indicating that masks can simultaneously communicate caution when worn and trustworthiness when observed [3,34,35].

Notably, before the COVID-19 pandemic, mask wearing was often interpreted as a sign of illness or heightened risk, contributing to social avoidance and psychological barriers [36,37]. Whether masks will eventually revert to their pre-pandemic connotations or continue to reflect new social meanings remains an open question worthy of continued investigation.

Gender differences further highlight the complexity of mask wearing’s relationship with IPD. As shown in Figure 3, female participants were significantly more likely to voluntarily wear masks than male participants (72% vs. 44%), consistent with research suggesting that women tend to perceive greater health risks and adopt protective behaviors more frequently [20]. In addition, female participants exhibited smaller IPDs when interacting with other females, whereas the IPDs across other gender combinations were relatively uniform (Figure 5). This may reflect gender-based comfort and affiliation cues that continue to shape spatial preferences, even in a post-mandate environment. Prior studies on social bonding and gender norms indicate that women often maintain closer IPDs with same-gender individuals [2,38,39], which may help to explain the reduced IPD observed in female–female interactions in this study.

Our results contribute to the growing body of literature on the long-term effects of pandemic-induced behavioral adaptations. Kühne et al. [8] demonstrated that face masks can have both prosocial and antisocial effects depending on the context. Our findings reflect this complexity: voluntary mask wearing was associated with larger IPDs, suggesting a tendency toward increased social distancing among mask wearers—potentially indicating elevated risk perception or a protective orientation. Conversely, encountering a masked target was associated with smaller IPDs, suggesting that masks may function as prosocial signals that convey trust and safety. This dual pattern implies that the social meaning of voluntary mask wearing may depend on the perspective—whether one is the wearer or the observer.

These observed differences in IPD suggest that the pandemic may have contributed to lasting changes in how individuals regulate physical proximity, especially in cultures where mask wearing has become normalized [19]. Previous studies indicate that prolonged shifts in IPD may be influenced by prior experiences with public health crises, reinforcing more cautious spatial behavior over time [13,14]. Cultural norms also play an important role in shaping post-pandemic protective behaviors. In East Asian societies such as Taiwan, mask wearing has long been a social norm and may influence both the decision to wear a mask voluntarily and individuals’ comfort in close interpersonal situations. These cultural factors should be considered when interpreting the generalizability of the present findings.

While this study offers valuable insights into how voluntary mask wearing relates to IPD, several limitations should be noted. First, although the use of an online simulation aligns with validated protocols in earlier research, it may not fully replicate the complexity of real-world social interactions. Dynamic factors such as facial expressions, movement, or environmental context are not captured in this format. The use of only two avatar targets—both with neutral expressions and standardized clothing—also limits the ecological validity, as characteristics like emotional displays, perceived attractiveness, or social status may influence IPD judgments. Additionally, although surgical masks were used consistently, research suggests that the mask color and type can elicit different psychological responses. Future studies could benefit from incorporating immersive virtual reality and a more diverse array of stimuli to enhance the realism and generalizability.

Second, the observational design of this study precludes causal inference. Participants were not randomly assigned to mask-wearing conditions; instead, their mask status was recorded upon arrival. While this naturalistic approach increases the ecological validity, it introduces potential self-selection bias, as mask-wearing decisions may be influenced by unmeasured factors such as risk perception, anxiety, past infection experiences, or regional background. These variables were not assessed. The study also lacked counterbalancing of mask conditions or matching between participant and avatar characteristics (e.g., gender or mask status), which limits interpretation regarding possible social mirroring effects. Additionally, although mask usage was monitored throughout the session, it was not strictly enforced or recorded during task execution. Finally, because the study was conducted in Taiwan—where mask wearing remains socially normative—the findings may not generalize to populations with different cultural or pandemic-related experiences. Future research should incorporate randomized designs, individual psychological measures, and cross-cultural comparisons to better understand how voluntary protective behaviors continue to shape interpersonal dynamics.

Understanding the long-term implications of voluntary protective behaviors is essential as societies adapt to the aftermath of the pandemic. While our results reveal meaningful associations between voluntary mask wearing and IPD preferences, these should be interpreted as correlational rather than causal. Future research should examine whether these behavioral patterns persist or diminish over time and explore how cultural, contextual, and individual-level factors—such as personality traits, perceived vulnerability, and previous health experiences—contribute to the adoption and maintenance of voluntary protective behaviors. Such investigations would offer deeper insights into the evolving relationships among public health practices, social norms, and risk perception in a post-pandemic society.

## 5. Conclusions

Our findings demonstrate that voluntary mask wearing remains significantly associated with IPD preferences in post-pandemic contexts. Female participants exhibited higher voluntary mask adoption rates (72% vs. 44%), and those who wore masks tended to maintain greater IPDs. In contrast, encounters with masked targets were associated with smaller IPDs, suggesting a nuanced dual role for masks in post-pandemic social interactions. These patterns offer evidence-based insights to inform public health strategies and the design of social spaces.

Public health authorities may benefit from recognizing the behavioral clustering observed among voluntary mask wearers, who tend to exhibit sustained protective behaviors. Given the substantial gender differences in mask adoption, gender-sensitive approaches should be considered in future public health messaging and intervention design. In spatial planning, social environments could be adapted to accommodate IPD differences—approximately 20 cm—between masked and unmasked interactions by incorporating flexible, modular layouts. Additionally, the observed social signaling effects—where masked individuals create a perceived zone of safety—can inform crowd flow and space allocation strategies in both public and private settings.

The shift from mandated to voluntary protective behaviors presents an opportunity to develop sustainable, choice-driven frameworks for future public health preparedness. Understanding how voluntary behaviors reshape social spatial norms is essential in creating culturally adaptive strategies that support community health resilience while respecting individual autonomy. For policymakers, these findings underscore the importance of integrating voluntary protective behavior patterns into pandemic response planning, implementing gender-sensitive public health measures, and designing adaptable environments that reflect the dual signaling effects of mask wearing in post-pandemic life. These quantifiable behavioral trends offer a practical foundation for evidence-informed social policy and spatial management in future public health contexts.

## Figures and Tables

**Table 1:** Results of four-way ANOVA on interpersonal distance.

Source	F	*p*-Value	η^2^
Participant gender (PG)	17.47	<0.05	0.022
Participant mask (PM)	35.29	<0.001	0.044
Target gender (TG)	1.09	0.298	0.001
Target mask (TM)	35.78	<0.001	0.045
PG × PM	1.86	0.173	0.014
PG × TG	4.52	<0.05	0.018
PG × TM	0.09	0.765	<0.001
PM × TG	0.34	0.559	<0.001
PM × TM	0.06	0.800	<0.001
TG × TM	0.01	0.909	<0.001
PG × PM × TG	0.01	0.906	<0.001
PG × PM × TM	0.40	0.527	0.001
PG × TG × TM	0.07	0.797	<0.001
PM × TG × TM	0.03	0.857	<0.001
PG × PM × TG × TM	0.01	0.924	<0.001

**Table 2:** Results of three-way ANOVA on interpersonal distance within each participant gender group.

Source	F	*p*-Value	η^2^
Male participants			
Participant mask (PM)	11.11	<0.001	0.028
Target gender (TG)	0.62	0.430	0.002
Target mask (TM)	20.93	<0.001	0.052
PM × TG	0.12	0.734	<0.001
PM × TM	0.08	0.782	<0.001
TG × TM	0.07	0.787	<0.001
PM × TG × TM	0.04	0.841	<0.001
Female participants			
Participant mask (PM)	25.41	<0.001	0.062
Target gender (TG)	4.78	<0.05	0.015
Target mask (TM)	15.38	<0.001	0.038
PM × TG	0.24	0.628	0.001
PM × TM	0.37	0.541	0.001
TG × TM	0.01	0.921	<0.001
PM × TG × TM	<0.01	0.954	<0.001

## Data Availability

The data are available upon reasonable request to the corresponding author.
